# A New Conceptualization of the Conscience

**DOI:** 10.3389/fpsyg.2018.01863

**Published:** 2018-10-08

**Authors:** Frans Schalkwijk

**Affiliations:** Department of Child Development and Education, Faculty of Social and Behavioral Sciences, University of Amsterdam, Amsterdam, Netherlands

**Keywords:** conscience, empathy, guilt, shame, morality, superego, neuropsychoanalysis

## Abstract

With the transition from a one-person psychology of instinctual needs to a two-person psychology of relational needs, the metapsychological focus tends to shift from instinct theory to emotion motivation and systems theory, and, accordingly, familiar concepts have to be rethought. In this article, the superego is reconceptualized as a psychic regulation system for self-evaluation, comprising the capacity for empathy, the proneness to experience self-conscious emotions, such as shame, pride, and guilt, and the capacity for moral reasoning. This new conceptualization provides useful tools for addressing the actual functioning of the conscience in clinical psychoanalysis. Affective neuropsychoanalysis can make important contributions to this rethinking of the superego. It also brings clinical practice and psychoanalytic metapsychology closer to empirical research beyond the scope of clinical psychoanalysis. The new model offers ample opportunities for integrating affective neuroscience into the functioning of the conscience.

## Introduction

As not only a practicing psychoanalyst but also a forensic reporter, I have a special interest in the conceptualization and workings of the superego. In my consulting room, I assess the influence of the superego in phenomena, such as self-punishment, a prohibition against rivalry and triumph, survival guilt, or (toxic) proneness to experience shame. Were I to diagnose the superego of these patients, I probably would not get much further than conclusions such as “a harsh superego” or “a punitive superego.” The same goes for my forensic practice: when having to diagnose the superego of a delinquent juvenile in a forensic report, I am at a loss for words, given that a comprehensive theory for describing the superego is lacking (Le Sage, 2005). As far as I know, research into the superego is reserved for scientists outside of psychoanalysis.

The clinical literature on the superego, however, is vast and theoretically diverse. Nowadays, we have a diverse body of knowledge on the superego and new publications continue to appear, with some expanding drive theory (Reddish, 2014) and others trying to integrate new theoretical developments pertaining to the relational turn, such as attachment theory (Holmes, 2011; Carveth, 2013). Nevertheless, after over a hundred years of psychoanalytic theorizing, the concept of superego has become a container concept^1^. It can refer to totally different concepts, ranging from an “ego-destructive superego” (Bion, 1959) to “a supervisor of the ego” (Blum, 1985), or to “a set of cognitive moral or ethical guidelines” (Brierley, 1962). It not only contains the ideal ego but also the ego ideal: a sense of longing for the future (Chasseguet-Smirgel, 1976). Accordingly, the birth of the superego has been situated across a wide swath of the child’s development: from the earliest months in Kleinian theory to the resolution phase of the Oedipus complex in Freudian theory around the age of six.

In my search for concepts that would offer not only a language that could describe clinical finesses but would also open up the possibility for operationalizing the superego, I found solid ground in the theories that appeared after the so-called “relational turn” in psychoanalysis and neuropsychoanalysis.

“Relational turn” refers to the transition over the past three decades in which psychoanalysts have turned away from the one-person psychology based on instinctual drives to the two-person psychology based on relational needs and systems for attachment, emotion regulation, or mentalization. Some of these regulation systems operate in the unrepressed unconscious (non-conscious implicit memory), others come into awareness when attention is turned to them, and still others have become conflictual and dynamically unconscious. Exemplary for this relational turn is when Meissner (2009a), in referring to the superego, writes: “Human motivations and emotions do not call for derivation from a putative drive” (o.c., p. 822). Influential authors have instead described emotional regulation systems which interact in a process of continuous unfolding affects, intentions, and goals (Panksepp, 1998; Akhtar, 1999; Fonagy et al., 2002; Schore, 2003; Jurist et al., 2008; Meissner, 2009a; Damasio, 2010; Lichtenberg et al., 2011; Panksepp and Biven, 2012). Many of these authors call for the reformulation of (part of) our existing metapsychology, something which would create new possibilities for integrating scientific knowledge from fields outside psychoanalysis, such as affective neuroscience or emotion theory.

In this paper, I hope to open up the discussion on the metapsychology of the superego. If we no longer assume that we are driven by sexual and aggressive drives but by affects, emotions, and feelings, how do I then conceptualize the superego? In “Reconceptualization of the ‘superego’ into ‘conscience,”’ I will propose that we no longer think in hypothetical mental structures, but rather that we approach the superego as a regulation system of the self. Then, in Section “Which psychic systems contribute to the functioning of the conscience?” I will describe three subsystems that participate in the functioning of the superego: the capacity for empathy, the proneness to experience self-conscious emotions, such as shame, guilt, and pride, and the capacity for logic, moral thinking. Finally, I will conclude that this new model for the superego can expand existing clinical knowledge, serve as the subject of research, and how a science like neuropsychoanalysis can expand our psychoanalytic knowledge.

It is not my intention to bypass or devaluate the existing body of knowledge, let alone do away with it–that would be throwing out the baby with the bathwater. On the contrary, the challenge is to stand on the shoulders of the predecessors and built onward. Hopefully, this new conceptualization will offer a new and challenging perspective on the functioning of the superego. After all, this reformulation is not an endeavor for its own sake; its purpose is to expand the possibilities for integrating new knowledge. My opting for a functional system instead of a mental structure can be traced back to the influence that neuropsychoanalytic theories have on my thinking. Structure implicates a causal, linear model of drives, and brain localization. (“Where is the superego situated in the brain?”). Systems theory has replaced causal thinking in almost every science and, in neurology, the brain is conceptualized as consisting of functional systems, with interacting brain regions co-creating specific mental phenomena (Damasio, 2010). In Section “Which psychic systems contribute to the functioning of the conscience?” I will describe how the conscience is assumed to be a psychic system in which different mental phenomena and their corresponding brain regions interact.

## Reconceptualization of the “Superego” Into “Conscience”

In what follows, I propose a reconceptualization from the superego as a “mental structure” to the superego as a “regulation system of the self.” For conceptual clarity, I will use “superego” when referring to the concept of superego in Freud’s structural and drive model and in object relations theories, and “conscience” when referring to the concept of superego based on the relational turn and neuropsychoanalysis. Of course, it is not the purpose of this paper to reduce the functioning of the conscience to brain functioning. Before describing the systems-view concept of the conscience, I will dwell shortly on the concept of self, as it is the self which is regulated by the conscience.

For conceptualizing the self in relation to the conscience, the work of Allan Schore and Daniel Stern on the nature of the self is of paramount importance.

Schore (2009) gives credit to Kohut, who broke new ground in psychoanalysis by turning away from an intrapsychic unconscious and a cognitive ego toward a relational unconscious and an emotion-processing self. Kohut, Schore cites, explored basic problems in psychoanalysis like “how do early relational affective transactions with the social environment facilitate the emergence of self (*development of the self*), and how are these experiences internalized into maturing self-regulating structures (*structuralization of the self*) (...)” (o.c., p. 190). According to Kohut, the self develops out of the interaction with the primary caretakers: the child internalizes the way his or her emotions are regulated in the interaction. “These regulating self-selfobject experiences provide the particular intersubjective experiences that evoke the emergence and maintenance of the self” (o.c., p. 192). Then, from a neuroscientific perspective, Schore provides answers to Kohut’s core problems, which Kohut was unable to answer in his time.

Central to Schore’s thinking is the notion that the idea of a single unitary self is misleading: “What we call the self is in reality a system of self states, that develop in the early years, but grow to more complexity during the life span” (Schore, 2017, p. 74). In the first year of life, the structuralization of the right brain self develops in the course of the interdependent interaction between child and caretakers (selfobjects), especially through processes of mismatch and repair in attachment, and with it (mal)adaptive implicit self-regulation processes develop. In early development, this implicit self, supposedly located in the lateralized right brain, is basically relational, as the self-states develop out of the interaction with the selfobjects. Schore (2009, 2017) locates the brain’s major self-regulatory systems in the orbital prefrontal areas of the right hemisphere. Its functioning belongs to the unrepressed unconscious; its content can be felt but cannot be translated into words or symbols. Accordingly, in psychotherapy, it cannot be reached through interpretations making the unconscious conscious, but it becomes visible in enactments between psychoanalyst and patient. For the functioning of the superego, it is important to realize that this right brain self responds faster to emotional stimuli than every other cortical function. These early developmental processes are required for the later “maturation of effective *superego* *autoregulatory systems”* (Schore, 2003, p. 151, italics FS). Somewhat later in early development after the second year, the verbal, conscious left lateralized self-system (“left mind”) develops. Schore writes: “Despite the designation of the verbal left hemisphere as “dominant” due to its capacities for explicitly processing language functions, it is the right hemisphere and its implicit homeostatic survival and affect regulation functions that are truly dominant in human existence” (Schore, 2017, p. 74). In Section “Which psychic systems contribute to the functioning of the conscience?” when discussing shame and guilt, I will discuss the implications of these notions for the functioning of the conscience.

From an attachment theory perspective, Stern (1985) describes that the mastering of emotions leads to an early developmental awareness of the self-as-agent, a repository of unconscious processes and actions. Around the second year of life, this self-as-agent is established. When, later in the development of the child, the quality of the important relationships has gradually been internalized into self and object representations, the self-as-subject develops, with the child experiencing affects and thoughts about its being and doing., Still later in development, the self-as-object begins to develop as a result of growing mental capacities, such as mentalization, emotion regulation, attachment patterns, and symbolic thinking. A firm sense of identity establishes itself when the theory of mind has been firmly established around the fifth year and the different modes of mentalizing have been integrated around the seventh year. Now, the conscious capacity for regulation of the self begins to develop: the self is continually evaluated against the backdrop of self and object representations. Once developed, the conscience cannot be switched off and on as one pleases, but is thereafter on stand-by until an emotion signals a threat to one’s self-esteem. And, as Lichtenberg et al. (2011) maintain, when this occurs defence mechanisms are activated almost without exception at the same time.

The function of the system “conscience” is to ensure that the self remains stable. In discussing consciousness, Solms (2017) argues that this striving for homeostasis of the self pertains specifically to “basic (brainstem) consciousness, which consists in *states* rather than *images*” (o.c., p. 6). This is the self-system Schore calls the implicit self, situated in the unrepressed unconscious. Despite this need for stability, in the reality of daily life the functioning of the conscience is nevertheless volatile: in some situations, the subject might be less empathic than normal, harsh in self-evaluations, or act out of line with his or her moral standards, while under different circumstances, he or she seems to evaluate him or herself in a neutral or even positive way. Intoxication due to drugs or alcohol alters how we experience self-conscious emotions, as do important life events, such as the birth of a child, divorce, or mourning for a lost parent.

## Which Psychic Systems Contribute to the Functioning of the Conscience?

**FIGURE 1:**
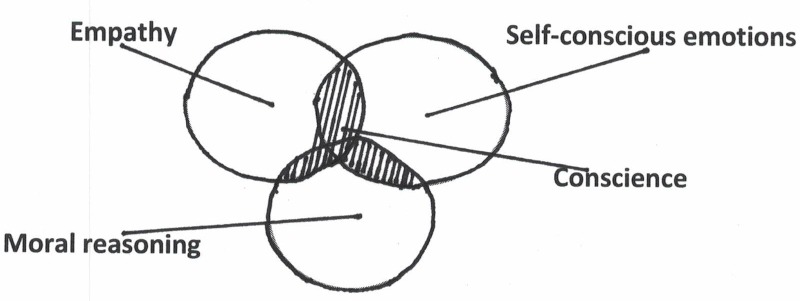
Functioning of the conscience.

Which psychic systems participate in regulating the self (see **Figure 1**)? In line with his definition of the implicit self as essentially relational, Schore (2009) calls empathy a core process of self psychology. Thus, the capacity for empathy is of paramount importance in the functioning of the conscience. The aspect of empathy in the evaluation of the self can take place *inter*psychically in factual interactions with others and *intra*psychically in interaction with internalized selfobjects, as well. The second system participating in the conscience is the proneness to experience self-conscious emotions, like shame, guilt, embarrassment, and pride. Feeling emotions are a signal that self-evaluation takes place, which the conscience is actively trying to restore homeostasis of the self. Finally, the functioning of the conscience is associated with the cognitive capacity for moral knowledge and reasoning. This cognitive ability functions as a cognitive backdrop against which self-evaluation takes place, not so much based on the interaction with others, but more on a cognitive, rational level of experiencing.

For conceptualizing the self in relation to the conscience, Daniel Stern and Allan Schore’s work on the nature of the self is of paramount importance. Based on the following, the reader might assume that I take a primarily neuroreductionistic stance, almost as if emotional life could be fully understood from brain functioning. This, however, is not what intend to emphasize. For me, the core feature of psychoanalytic thinking is the developing child imparting meaning to affects, emotions, and feelings, which ultimately crystallize into the adult identity. Thus, an important theoretical aspect of psychoanalysis is it being grounded in phenomenological science. Since the relational turn, psychoanalysis has also become a science about intersubjectivity, i.e., about the subject imparting meaning in a relational context. And, lastly, affective neuroscience can enlighten the neurological correlates of our subjective states.

### Empathy

In Section “Reconceptualization of the ‘superego’ into ‘conscience’,” we saw that the first self-experiences take shape in attachment and emotion regulating processes between caretaker and child, leading to an implicit self that is basically relational. It is thus obvious that, from a developmental perspective, the capacity for empathy is the first mental activity that triggers evaluation of the self. Empathy is not an emotion or feeling itself, but a specific mental activity, closely associated with mentalising but differing from it (Allen et al., 2008). It is being able to adequately experience the emotion of another as separate from one’s own emotions (Bolognini, 2004; Aragno, 2008). Empathic activity is dominated to a greater or lesser extent by cognitions or by feelings, leading to the conceptual differentiation between cognitive and affective empathy (Hoffman, 2000; Meissner, 2009b). In the reality of daily emotional life, empathic activity is naturally often a mixture of the two: empathy is a layered phenomenon, a synthesis of knowing the other’s perspective and feeling the compassion to help.

In contrast to the functioning of the conscience, the empathic capacity can be more or less consciously switched on and off or function in many other in-between states. Being angry at someone lessens the quality of empathy, sometimes as much as does being in love. Empathic activity is undermined by shame, as shame can evoke an inward directed emotion, whereas empathy is other directed (Schalkwijk, 2015). In our clinical work, we meet both patients who have developed a rigid empathic wall to protect themselves from being overwhelmed by others’ emotions and patients suffering from empathic anger who react as though they themselves are the victim (Nathanson, 1986). Normally, the functioning of an empathic wall helps the subject not to be overwhelmed by the other’s emotions. In borderline pathology, this function of the empathic wall falls short: the wall is so to speak “too thin.” The quality of the empathic activity is highly influenced by a wide array of variables, such as the closeness of the other (family, partner, friend, acquaintance, belonging to the same religious group, and race), the likeability of the other, and many other variables (Hoffman, 2000; Watt, 2007).

Of the three domains of the conscience, empathy has been researched most. The functioning of mirror neurons is being hyped as the neural substrate for empathy; however, mirror neuron pioneer Gallese (2008) warns strongly against this simplification. Neuropsychoanalytically, one would expect empathy to be associated with the right brain implicit self-system, leading to empathic processes which take place in implicit, non-verbal communication, while the left right brain self-system is associated with more cognitive, conscious processes. Fonagy et al. (2002) have indeed proposed the existence of two separate Internal Interpretative Mechanisms (IIM) for interpersonal understanding of emotions and belief states. The cognition-focused IIM (IIM-c), associated with the theory of mind is characterized by activation of medial prefrontal foci around BA8, whereas the affect-focused IIM (IIM-a) is associated with the amygdala and orbitofrontal areas. In experiments with face recognition, affective empathy involves a primitive, automatically activated, fast-firing neural circuit functioning alongside a more developed, cognitive, and relatively slow-firing circuit (Adolphs, 2002).

How do these brain processes contribute to the functioning of the conscience (Blair, 2003; Decety and Meyer, 2008; Baron-Cohen, 2011)? Fonagy et al. (2002), (p. 139) hypothesized the following: “Individuals with limited capacity for empathy will show limited responses to the depiction of distress in children’s faces, while others will show no amygdala deficit but demonstrate limited OFC activation. Those with amygdala impairment are likely to have failed to acquire a proper understanding of their own emotional responses that directly led to a failure of empathy. (...) A third level of failure may occur as a consequence of a disconnection between the medial frontal and the orbital frontal areas (IIM-a and IIM-c).” Their hypothesis is validated by, among others, Decety et al. (2013): perpetrators with strong psychopathic traits are able to adequately sense the other’s pain, but they are not inhibited by it. Interestingly, brain circuits that are involved in discerning another’s pain overlap brain circuits involved with moral reasoning, emotions, and decision making.

### Self-Conscious Emotions^2^

In the same way, regulation systems for basic emotions are relationally based and developed, the regulation systems for self-conscious emotions can have an *inter*personal and an *intra*personal quality. Self-conscious emotions, however, are not biologically bestowed like Panksepp’s affective motivational systems, but rather they arise as a result of relatively complex mental processes and are less imbedded in biologically determined action tendencies than basic emotions (Tracy et al., 2007). They develop out of the experience of reflecting upon oneself as an object (“self-as-object”), something which can result in shame, guilt, pride, or embarrassment. Self-conscious emotions can appear in relation to the strictly individual self and to who someone is in a relational, social, or cultural context (Hoffman, 2000). They come into play when the subject acknowledges that an upcoming emotion, foretold by physical affects, does relate to one’s identity: “This has to do with me” (Mills, 2005). Once the emotion has been owned as pertaining to identity, consciously or unconsciously, it has to be regulated as it touches on the self-as-object. Empirical research has shown that people differ enormously in their proneness to experience self-conscious emotions (Tangney and Dearing, 2002). Some individuals genuinely lack any self-consciousness (Hare, 1999), which is a sign of pathology. Others suffer greatly from excessive guilt or shame (Seidler, 2000; Lansky, 2005; Erreich, 2011). Nathanson (1992) developed a clinically interesting Compass of Shame, which features four different styles for coping with shame: attacking the self, avoidance, denial, and attacking the other.

Describing early superego development, Schore (2003) has written extensively on affecting regulation in the service of continuity of the self, focusing strongly on shame in attachment experiences: “It is this moment of reunion of the “returning,” highly aroused, elated, practicing toddler, in a state of excited expectation, reconnecting with the mother, that is the prototypical object relation in the emergence of shame” (o.c., p.158). Schore points here to the importance of shame stress and the neurophysiology of arousal dysregulation during practicing reunion episodes, which are recognizable in adult life: “The brake of incremental arousal seen in shame (...) reflects a sudden dynamic switch from sympathetic dominant to parasympathetic dominant ANS activity” (o.c., p.162). Guilt is prominent in later (oedipal) development and might be associated more with the “later maturation of the linguistic rational capacity of the verbal analytic left hemisphere” (o.c., p. 185). Remember that Schore linked the implicit self-system to the lateralized right hemisphere and the explicit self-system to the lateralized left hemisphere.

Focusing on the relation between shame, guilt, and neurobiology, Schore (2003) writes: “(...) the shame system that emerges in this period represents an involving cortical inhibitory control mechanism of excessive, hyperstimulated states” (o.c., p. 158) and “Utilizing a neuropsychoanalytic perspective, it is suggested that the psychoanalytic ego ideal can be identified as an affect-regulation structure with the left orbital prefrontal cortex. This cortical inhibitory system is expanded in the right hemisphere and has extensive limbic connections, regulates emotions (...), attachment behavior (...), and aggression (...), and influences parasympathetic and sympathetic autonomic function.” (o.c., p. 182). Then turning to guilt, Schore concludes: “The earlier development of nonverbal shame and the ego ideal before verbal guilt and conscience reflects the known biologically determined earlier differentiation and functional onset of the nonverbal visuospatial-holistic right hemisphere (...) and the later maturation of the linguistic-rational capacity of the verbal analytic right hemisphere” (o.c., p. 185).

In a (non-psychoanalytic) review of the studies into the neural correlates of shame and guilt, Bastin et al. (2016) found distinct patterns of brain structure/function for these emotions^3^. Shame was more likely to be associated with activity in the dorsolateral prefrontal cortex (dlPFC), posterior cingulate cortex, and sensorimotor cortex function. Bastin et al. hypothesize the dlPFC-activity to be an indication of the “harder mental work” needed to regulate the negative affect in comparison to guilt. The fact that the activity in the anterior cingulate cortex is involved in the processing of reward and punishment in shame but not in guilt might point to the experience of shame as the self is being threatened. This finding is in line with the notion that shame pertains more to an evaluation of identity and guilt pertains more to one’s own actions (Lewis, 1971). Guilt was more likely to be associated with activity in the dmPFC, the ventral anterior cingulate cortex (vACC), the posterior temporal regions including the temporoparietal junction (TPJ), the precuneus, and the premotor cortex. Activity in the TJP is associated with shifting attention and orientation, and the workings of the theory of mind (recognizing the other’s intentions and realizing false beliefs). The precuneus as part of the limbic system is associated with emotions, with a feeling of self. And, with respect to guilt, the dmPFC-activity is associated with self-referential processing, self-focused cognition, and theory of mind, which ties in with Schore’s explicit self: feeling and knowing to be a self. Etkin et al. (2011) hypothesize that vACC-activity is associated with emotion regulation by facilitating planning of adaptive responses, one of the positive qualities that has been ascribed to guilt in the literature on shame and guilt. This finding is in line with shame and guilt theory, namely that guilt pertains to one’s action, and enables repair and reconstruction (Lewis, 1971). Finally, Bastin et al. (o.c., p. 467) argue that activity in the TPJ and posterior MTC/STS “is in line with these regions to TOM, given that guilt has been particularly linked with other-processing including reading other’s state of mind/feelings/thoughts.” Here, we see how guilt and empathy do indeed converge in the functioning of the conscience.

### Moral Reasoning

People have moral beliefs that play a role in the evaluation of the self-as-object. In the psychoanalytic literature, the superego is the place where the internalized parental does and does not are found. It is tempting to restrict the moral domain to abstract thought, but the moment morality is related to “Who am I?” self-conscious emotions also come to the fore, especially when the acute awareness of “Who am I?” is in conflict with “How I thought myself to be.” In the course of cognitive development, a child acquires a wide array of social and moral norms. In the introduction, I mentioned already Brierley’s (1962) description of the superego as a set of cognitive moral or ethical guidelines.

On the bioneurological level, moral decision making has been associated with right vlPFC-activity: “The anterior temporal lobe is suggested to play a role in social conceptual knowledge, which is required to understand social concepts and rules (...) and to be aware of situations that are likely to elicit moral emotions (...)” (Bastin et al., 2016, p. 466). This activity is also associated with guilt, but not with shame, perhaps indicating more cognitively laden processes. The neural substrates of (a lack of) morality have also been extensively studied, especially in the field of forensic sciences (Cima, 2016). Moral reasoning has been associated with the posterior superior temporal sulcus, amygdala, insula, ventromedial prefrontal cortex (vMFC), dlPFC, and medial prefrontal cortex (Decety and Cowell, 2014). Raine and Yang (2006) argue that there is an overlap between these brain regions and those associated with antisocial behavior. An underdeveloped empathic capacity in psychopathy, for example, has been attributed to misfunctioning of the amygdala and the vMFC; one’s own behavior is not inhibited when seeing the other’s pain (Blair, 2003).

The contribution of hormones like cortisol, testosterone, and oxytocin to the functioning of the conscience is another interesting field of research (Fragkaki et al., 2017). For example, oxytocin seems to enhance the quality of empathy in subjects with a secure attachment style, whereas it might even disturb and affect regulation in subjects with insecure attachment (Decety, 2011).

Research into hormone levels and brain functions is important with respect to, among other things, answering the question of which biological functions serve to facilitate transgressive violence. For example, it appears that individuals with lower cortisol levels have fewer signals of physical arousal in tense situations (Popma, 2006). Normally, the thought of violent behavior is accompanied by an increase in physical tension, which potentially leads to one recoiling from acting out the thought; the body sets a boundary, as it were. For those who feel less tension, however, it is easier to turn to violence.

## Testing Hypotheses

The model provides an opportunity to bring clinical practice, psychoanalytic metapsychology of the conscience, and empirical research into the conscience closer together. As discussed in the Section “Introduction,” a conceptual language for the descriptive diagnosis of functioning of the conscience in forensic evaluation is lacking. In two studies, we put the new model to test by comparing offenders with non-delinquents (Schalkwijk et al., 2016; Verkade et al., unpublished). As might be assumed, our hypothesis was that offenders would score lower on all three domains of the conscience. We used questionnaires that measure empathy, proneness to experience self-conscious emotions, coping styles for shame regulation, and moral development. Contrary to our expectations, the two groups did not differ in an all-or-none fashion with respect to the development of the conscience. However, we could still differentiate between the groups in a clinically meaningful way: the offenders showed a relative lack of developmental ripeness on all three components of the conscience.

Neurobiological research on self-conscious emotions is only slowly developing and scarce, despite the important relationship between, for instance, shame on one hand and mental or physical health on the other hand (Dickerson et al., 2004; Morita et al., 2008). In a research project on shame and sleep disturbances, we induced a novel shame experience in participants by unexpectedly confronting them with a recording of their own singing, often out of tune, recorded without their knowing during a prior karaoke session, showing that for subjects with insomnia disorder shame experiences might contribute to a permanent state of hyperarousal and rumination (Wassing et al., 2016). In a follow-up study, shame experiences from the distant personal pasts of patients were re-evoked. Ongoing fMRI analyses comparing new and old shameful experiences suggest that good sleepers reorganize brain activity to make memories of shame less distressing. In contrast, when people with insomnia recall shameful experiences from the distant past, their brain activates as though these are a new distressing experiences that have just happened. This finding has important clinical consequences, as it shows that being in psychoanalysis or psychotherapy—which is a shame-evoking situation—might activate recollections of old shameful states and thus bring some patients into a state of non-amenable arousal.

## Conclusions and Questions

The advantages of this new model of the conscience are numerous.

Conceptually, it opens a new language for researching the connection between the self and the regulation of the self. Self and conscience are both regulation systems and thus functionally similar. Above, I suggested that the self regulates basic emotions and the conscience self-conscious emotions, but from a systems perspective, these regulation functions are probably co-dependent. Following Schore’s differentiation between the implicit right and the explicit left self-system, does the conscience follow a similar developmental course and is the conscience also to be understood as consisting of a system of consciences? More research is needed to address these questions.

Clinically, the new model opens up a language for operationalizing the functioning of the patient’s conscience, i.e., when he or she is experiencing self-conscious emotions. Schore showed that material from the unrepressed unconscious can only be expressed in enactments between patient and thus technically, asks for a different therapeutic approach than for material from the repressed unconscious (Schore, 2017). According to emotion regulation theory, this approach enhances the integrative capacity for dealing with the uncovered content (Schore, 2003). The new model broadens the scope of addressing the influence of the conscience to a broader listening. After all, a psychoanalysis is first and foremost a place where the focus is on the self-as-object. All interventions, however, respectfully they may be intended, can be experienced by the patient as an attack on his or her self-esteem (Akhtar, 2013). In therapy, inevitably, the self-regulation must be disturbed for change to take place, and thus the conscience is activated. In addition to thinking in the well-known triangle of “resistance, anxiety, and hidden impulse,” we could think also in the triangle of “resistance, fear for negative self-conscious emotions and low self-esteem.”

As this paper lays out, the model offers ample opportunities for integrating affective neuroscience in the theory of the conscience. After all, empathic activity, experiencing self-conscious emotions, and moral thought and behavior have corresponding neurobiological substrates: the brain has subjective states (Solms and Turnbull, 2010). It is fascinating to imagine how the new model of the conscience could open up possibilities for rethinking the workings of the superego. How can we theoretically understand and operationalize the well-known clinical phenomenon of shame undermining empathic activity: when shame is experienced, does its neural activity interfere with neural activity for system IIM-c or IIM-a for empathy? And does this effect only occur in subjects who prefer to use internalizing shame coping styles and/or is this effect absent in subjects who use externalizing shame coping strategies? And what about toxic shame, so often encountered in victims of abuse? How can we understand their disastrous attack on the self in terms of self-regulation with correspondent neuropsychoanalytic correlates? The model also provides opportunities for understanding why the functioning of the conscience is volatile and indeed highly individual. An offender might not feel shame or guilt when stealing from a stranger, but might experience these self-conscious emotions when stealing from his or her own father. In the former situation the distance to the victim is less likely to evoke empathy, whereas in the latter empathy is hard to avoid.

## Author Contributions

The author confirms being the sole contributor of this work and approved it for publication.

## Conflict of Interest Statement

The author declares that the research was conducted in the absence of any commercial or financial relationships that could be construed as a potential conflict of interest.

## Footnotes

1 In earlier drafts of this paper, references to “earlier” literature on the superego invariably led to the comment “But that’s not how this concept should be interpreted! You’re wrong.” Not being able to avoid such discussion, I leave out the earlier literature, although they form the solid ground my newer ideas are founded on. Drive theorists might object that my paper is another attempt to do away with what is fundamentally psychoanalytic and state that it seems like old wine in new bottles. That is fine with me, as since the introduction of the cap, the chance of tasting corked wine has been reduced immensely. So, I still will go for old wine in new bottles.

2 For many psychoanalysts, the concept of “self-conscious emotions” is probably unfamiliar. However, this concept is widely used in academic psychology when referring to emotions such as shame, guilt, pride or embarrassment (Tracy et al., 2007), as opposed to basic emotions such as hunger, thirst, anger or interest. Psychoanalytic colleagues who read earlier versions of this paper interpreted “self-conscious” too literally, as if the fundamental psychoanalytic idea of the existence of an unconscious mental life would be lost. This is not what I intend. In this article, “self-conscious” simply means “pertaining to the self,” regardless of whether it is conscious, dynamically unconscious or non-conscious in implicit memory.

3 In their review, the authors also refer to embarrassment, but for going into that emotion would ask for a theoretical discourse on the difference between shame, embarrassment, and guilt, which goes beyond the cope of this article.
